# Curvature and van der Waals interface effects on thermal transport in carbon nanotube bundles

**DOI:** 10.1038/s41598-022-22641-y

**Published:** 2022-11-14

**Authors:** Mostafa Valadkhani, Shunda Chen, Farshad Kowsary, Giuliano Benenti, Giulio Casati, S. Mehdi Vaez Allaei

**Affiliations:** 1 School of Mechanical Engineering, College of Engineering, University of Tehran, Tehran, Iran; 2 Department of Civil and Environmental Engineering, George Washington University, Washington, DC 20052 USA; 3 Dipartimento di Scienza e Alta Tecnologia, Center for Nonlinear and Complex Systems, Università degli Studi dell’Insubria, via Valleggio 11, 22100 Como, Italy; 4 Sezione di Milano, Istituto Nazionale di Fisica Nucleare, via Celoria 16, 20133 Milan, Italy; 5 NEST, Istituto Nanoscienze-CNR, Piazza S. Silvestro 12, 56127 Pisa, Italy; 6 International Institute of Physics, Federal University of Rio Grande do Norte, Campus Universitário-Lagoa Nova, CP. 1613, Natal, Rio Grande do Norte 59078-970 Brazil; 7 Department of Physics, University of Tehran, Tehran, 14395-547 Iran; 8 School of Nano Science, Institute for Research in Fundamental Sciences (IPM), Tehran, 19395-5531 Iran

**Keywords:** Condensed-matter physics, Structure of solids and liquids, Carbon nanotubes and fullerenes

## Abstract

A van der Waals (vdW) heterostructure, can be used in efficient heat management, due to its promising anisotropic thermal transport feature, with high heat conductance in one direction and low conductance in the rest. A carbon nanotube (CNT) bundle, can be used as one of the most feasible vdW heterostructures in a wide range of nanoscale devices. However, detailed investigations of heat transport in CNT bundles are still lacking. In this paper, we study heat transport in different CNT bundles—homogeneous bundles consisting of the one CNT radius (curvature) and inhomogeneous bundles constructed from different CNTs with different curvatures. We also investigate the comparison between two possible thermostatting configurations: the two ends connected (TEC) case in which there is at least a direct covalently connected path between the hot and cold heat baths, and the one end connected (OEC) case in which the system can be divided at least into two parts, by a vdW interacting interface. Nonequilibrium molecular dynamics simulations have been carried out for a wide range of configurations and curvature differences. We find that, in homogeneous bundles, by increasing the number of outer CNTs, the heat conductance increases. In inhomogeneous bundles, the total heat flux shows dependence on the difference between the curvature of the core and outer CNTs. The less the difference between the curvature of the core and the outer CNTs, the more the thermal conductance in the system. By investigating the spectral heat conductance (SHC) in the system, we found that a larger curvature difference between the core and outer CNTs leads to a considerable decrease in the contribution of 0–10 THz phonons in the bundled zone. These results provide an insightful understanding of the heat transport mechanism in vdW nano-heterostructures, more important for designing nanoelectronic devices as well as systems in which asymmetry plays a significant role.

## Introduction

van der Waals (vdW) heterostructures have attracted a lot of attention^1–4^ due to their applications in making novel structures with distinct optoelectronic and thermal characteristics^5^. The emergence of new vdW heterostructures brings exciting applications in different branches of science and technology, boosting an effort to understand their stable properties, different from those of pristine individual components^6^.

More specifically, concerning different common structures that can be produced in the synthesis of *aligned* nanotubes^7^, well known as nanotube forest, a common possible feature in the growth of nanotubes on a large scale area, vdW interaction between nanotubes and the number of neighbors and structures become more important. In spite of numerous studies that have been devoted to these kinds of structures in different research areas, such as thermionic emission and plasmonics^7,8^, or free carriers in aggregated single-wall carbon nanotubes by photoexcitation^9^, microelectromechanical devices^10^, etc., thermal transport in these different structures has not been well studied, yet.

In general, mechanical stability, electrical and thermal properties of CNTs make them appropriate candidates for optimal thermal management of the nanoscale devices^11–13^. Considerable efforts have been devoted to finding different aspects of thermal properties of CNTs^14–17^, but for the vdW heterostructures, further investigations are highly required.

Thermal stability and heat management are two leading parameters to increase the performance of electronic devices^18,19^. In particular, proposing or synthesis of materials or structures with anisotropic thermal conduction can be useful from different points of view^17,20^. For instance, how to remove high temperature from a hot spot along the direction of high thermal conductivity as well as provide kind of insulation in the other directions^21^, even leading to the design of a thermal rectifier^17^. The most well know example is multiwalled CNT, due to the strong covalent bond inside the tubes and, in comparison, weak vdW interaction normal to CNT surface Due to the various configurations in possible structures of CNT bundles, anisotropic thermal transport features, due to the structure, shape, and curvature, can be promising.

**Figure 1:**
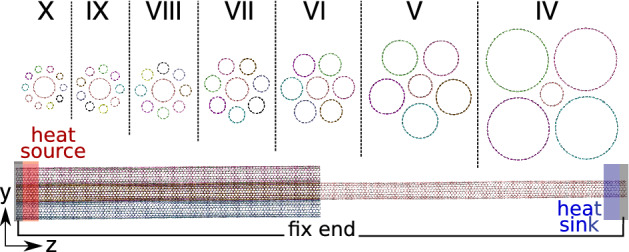
Schematic structure of bundled CNTs with different diameters (top). The configuration of the system, consisting of two heat baths and the two ends, the region in which the heat baths are implemented, and *z* axis corresponding to heat flux direction and the other axes (*x* and *y* normal to energy flux direction, (so-called radial direction) are presented. The two red (heat source) and blue (heat sink) regions are corresponding to the hot and cold heat baths.

In this paper, we study the thermal transport in different bundles of CNTs, characterizing the effect of different curvatures, as well as the contact area. This study leads us to understand thermal transport dependence of most common possible configurations of CNT bundles with respect to their curvature. More specifically, we consider a structure consisting of a CNT as *the core*, and a number of first neighbors, i.e. *the outer CNTs* (from 3 to 10), see Fig. 1 for a schematic drawing. Using non-equilibrium molecular dynamics (NEMD) simulations, and spectral analysis, heat transport properties such as heat fluxes and Kapitza resistance are calculated. Depending on the number of outer CNTs, different CNT diameters, the spectral thermal transport is studied. In cases that the radius of outer CNTs differs from the core one, the effect of different curvatures plays as another key parameter in thermal transport, which is also investigated here. To gain a better understanding of heat transport properties, we use tools such as the power spectrum and spectral heat conduction (SHC). Regarding the two different important parameters that play roles in heat transport of CNT bundle structure, the rest of the paper is organized as below. In “Simulation method”, we explain how different structures are made, and details of NEMD have been explained. The results and discussion section has been divided into two parts; the section in which the core and the outer bundles are the same, in which we address the importance of a shared interface between CNTs. We also study the effect of the difference in curvature of the core and outer CNTs and its effect on spectral heat transport in the rest of the section.

## Simulation method

**Figure 2:**
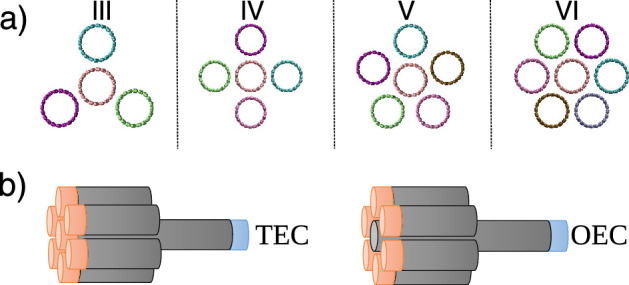
(**a**) The bundle structure for cases that the core and outer CNTs have the same diameters (i.e. (10,10)). The structure of the system, such as the length, position of fixed atoms, and heat baths are the same on the rest of the simulations. A case with III, IV, V, and VI outer CNTs have been schematically shown. (**b**) Schematic of tow ends connected core (TEC) and one end connected core (OEC) in which the blue and orange colors represent heat sink and heat source respectively.

In a given CNT vdW heterostructures, from nanotube forest^7,8^ to self-assembled ones^9,10^, a given local bundle of CNTs can contain a different number of adjacent tubes, with the same diameter (see Fig. 2a) or probably with smaller or larger ones (see Fig. 1). Here we have to consider a wide range of possible bundle structures, to have a good estimation of key parameters, as well as be able to observe leading parameters in heat transport features. The atomistic structure of bundled CNT is schematically shown in Fig. 1. There are seven different structures IV, V, VI, VII, VIII, IX, and X that are corresponding to the number of outer CNTs surrounding the core one. The (*m*, *m*) chirality is chosen for all CNTs in which *m* is 27, 15, 10, 7, 5, 4 and 3 for IV, V, VI, VII, VIII, IX, and X case, respectively, that it means that *m* is 10 for the core all CNTs in homogeneous structures. Diameter of outer CNTs have been chosen in such a way that all CNTs become perfectly matched with each other in a given bundle structure, i.e. the distance between each adjacent CNTs to be almost equal to the distance between graphite sheets, we call it as *vdW equilibrium distance*.

**Figure 3:**
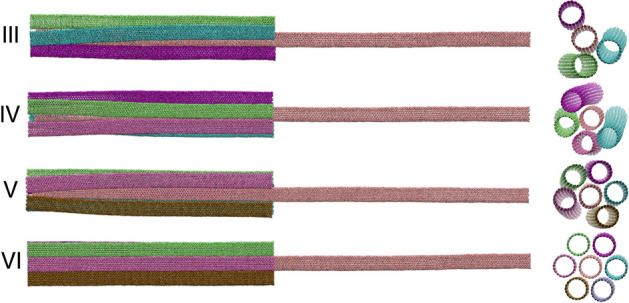
If a few atoms of outer CNTs are not fixed, rearrangement can be obtained in III, IV, and V cases. A sample snapshot of these systems has been presented. Just in VI case, there is no deviation.

As common and possible cases, Fig. 2a, we also study the heat transport in structures in which the diameter of outer CNTs are the same as the core ($m=10$), so the *vdW equilibrium distance* of outer CNTs does not comply in structures other than I-VI in which six CNTs with the same diameter of inner CNT are surrounding the inner CNT as shown in Fig. 2a, different from what is considered here, as vdW equilibrium distances. In these cases, the distances between the outer CNTs can vary from the I–VI case, the structure could be unstable and it is important to consider this effect in computing transport properties. In Fig. 3, a sample possible structure of the system has been shown. It means in addition to molecular fluctuations, these structures could have additional reformation by which the distances between outer CNTs can change. This can affect the phonon transport in every individual CNT, and a possible additional curvature along the CNT axis also can affect thermal conductivity. To assure to have not a sample-dependent study for these cases, where the diameter of the core and outer CNTs are the same, by imposing additional forces on a few atoms in outer CNTs, we eliminate the reconfiguration and rearrangement of studied structures. Thus, what we have investigated in these structures can be mostly similar to the structures that are presented in Fig. 2a.

All molecular dynamics simulations of this study are performed using the LAMMPS code^22^. Airebo potential^23^ is used to simulate C–C interactions. Airebo potential is suitable for simulating thermal transport in carbon nanotubes as shown in different studies^17,24–26^. (To support the independence of our main findings from the covalent C–C potential, in the supplemental materials we compare results of the main text with those obtained by means of the optimize Tersoff potential^27,28^ with the same van der waals (LJ) parameters used in the AIREBO potential.) Stuart et al. ’s Lennard Jones potential parameters^23^ is used to simulate the interactions between each CNT pair. The time-step in velocity Verlet integration is set 1 fs. The core CNT length is 50 nm and the outer ones are 25 nm. Each steady heat current in each stable structure has been obtained by imposing the procedure. At the first, an isothermal-isobaric (NPT) ensemble imposed for $5\times 10^5$ steps (0.5 ns) at 300*K*, and afterward $3\times 10^5$ steps (0.3 ns) in canonical (NVT) ensemble lead each system to achieve equilibrium condition. Then $1\times 10^5$ steps (0.1 ns) in the microcanonical (NVE) ensemble are performed to reach a state that we could impose two heat baths on different sides of each system. Atoms in the two slabs with the length of $10 \, \text{\AA}$ at both ends of each structure are frozen to have fixed boundary conditions at both ends. Atoms in two slabs with a width of $20\, \text{\AA}$ adjacent to the frozen regions are coupled to a Langevin thermostat. The heat source and sink temperatures are set to 350 K and 250 K, respectively.

We have used Langevin thermostat^29,30^ to fix temperature in heat baths to reduce the artificial effect on temperature profile^31^. The importance of this heat bath type is discussed in “Results and discussion”. For $5\times 10^6$ steps (5 ns), the two heat baths are imposed to reach ${\Delta {\rm T}}$. Then another $1\times 10^6$ steps (1 ns) perform to reach the steady-state and finally $3\times 10^6$ MD steps (3 ns) are carried out to calculate temperature profile and collect velocities of atoms every 5 time steps to calculate spectral heat conductance (SHC).

To compare the frequency dependence of heat transport, mostly have an insight on which modes are more contributing in the core, and how vdW interaction can improve/suppress the transport inter- and intra-tubes, we calculated the spectral heat conductance of the core. The well known formalism developed by Saaskilahti et al.^32^ has been employed for calculating spectral decomposed thermal conductance or SHC. The SHC is calculated for two cross-sections in the middle portion of the single side and the bundle side of the core (orange triangle and blue square in schematic in Fig. 5). To calculate SHC, we need to measure $q_{i\rightarrow j}(\omega )$, the interatomic heat current between particles *i* (in the left side of the cross-section) and *j* (in the right side of the cross-section) located next to each other, by which it is possible to express the pair-wise SHC equation^32^

1 $$\begin{aligned} q_{i\rightarrow j}(\omega )=-\frac{2}{t_{simu}\omega }\sum \limits _{\alpha , \beta \in \{x,y,z\}} \text {Im}\langle {\hat{v}}^\alpha _i(\omega )K^{\alpha \beta }_{ij}{\hat{v}}^\beta _j(\omega )\rangle , \end{aligned}$$

where $t_{simu}$ is the simulation time, $\omega$ is the angular frequency, ${\hat{v}}^\alpha _i(\omega )$ and ${\hat{v}}^\beta _j(\omega )$ are the discrete Fourier transforms of the atomic velocities, and the interatomic force constant $K^{\alpha \beta }_{ij}$ can be written as

2 $$\begin{aligned} K^{\alpha \beta }_{ij}=-\frac{\partial ^2U}{\partial u_i^\alpha \partial u_j^\beta }\bigg |_{u=0}, \end{aligned}$$

in which, $u_i^\alpha$ and $u_j^\beta$ are the displacements of atoms *i* and *i* from their equilibrium positions in directions $\alpha ,\beta \in \{x,y,z\}$, and *U* is the interatomic potential energy function. The SHC through the core of is obtained from Eq. (1), by a summation over all pairs of atoms (one in from left outer CNTS, denoted by ${\mathfrak {L}}$ and one from the core, denoted by ${\mathfrak {R}}$) within the potential cut-off distance of each other and dividing by the interface area *A*:

3 $$\begin{aligned} q(\omega )=\frac{1}{A}\sum \limits _{i\in {\mathfrak {L}}}\sum \limits _{j\in {\mathfrak {R}}}q_{i\rightarrow j}(\omega ). \end{aligned}$$

## Results and discussion

In a forest or an assembled structure of CNTs, different local structures can be obtained. Among all possible configurations, a bundle structure is one of the most possible, with orientational ordering. For example, one local bundle can have a core tube with a few to several adjacent CNTs, possibly with the same (homogeneous) and/or different (inhomogeneous) diameters. To clarify all differences with respect to heat transport, here, all cases have been categorized into homogeneous (Fig. 2a) and inhomogeneous (Fig. 1) local structures.

In a homogeneous case in which the vdW equilibrium distance between the core and the outer CNTs are the same, the most important issue is the number of neighboring CNTs (Fig. 2a). The higher the number of neighbors, the larger the shared surface area among tubes. A larger area between two neighboring CNTs corresponds to larger vdW interaction between them, which here is addressed as the *shared vdW surface* in heat transport in a CNT bundle.

On the other hand, in an inhomogeneous case (Fig. 1), each element of a bundle can have a different diameters, thus despite the importance of the shared surface, the curvature difference between each element can also play a significant role. The effect of interface shape or diameter difference between two neighbor CNTs, here, has been categorized as the *curvature effect*. Thus, this section is divided into two parts to address both issues.

From another point of view, there are two different heat transport scenarios in these kinds of heterogeneous vdW structures. In the first case, there are combinations of vdW and covalent interactions that are simultaneously contributing to heat transport. But, in the second scenario, there is no direct path from hot to cold heat bath that is constructed by covalent bands; thus all phonons need to pass through the shared surface between outer CNTs and the core, via vdW interaction. Thus, these two cases have been divided into two different categories: *Two End Connected (TEC)*, when the core CNT is in contact with both heat baths, in which a portion of heat fluxes cross directly from the heat source to heat sink through the core CNT, and *One End Connected (OEC)* when the core is just in contact with the cold heat bath from the right side, while the hot heat bath is in contact with the outer CNTs (from the left) as shown in Fig. 2b. In this case, the entire heat flux crosses the interface between the core and the outer CNTs.

**Figure 4:**
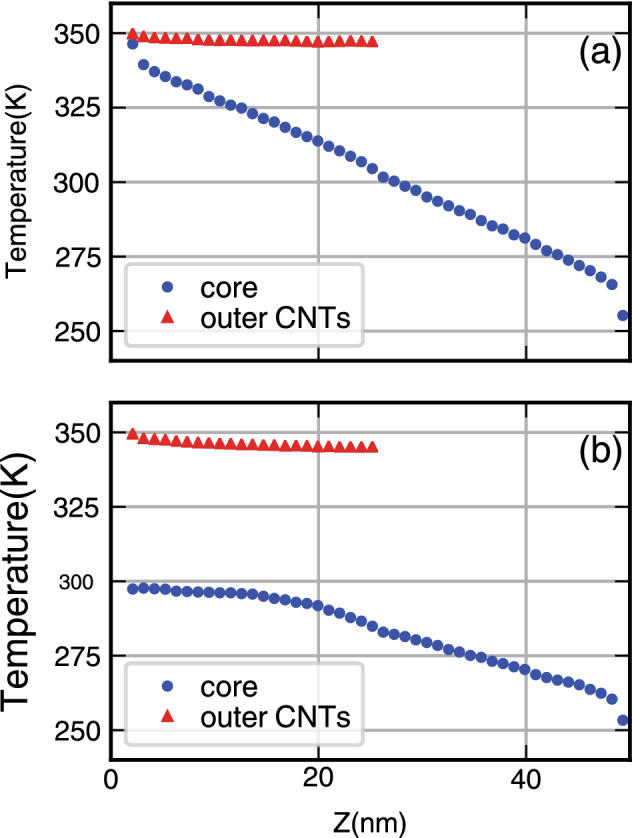
Temperature profiles for the core (blue) and the outer CNTs (red) for (**a**) TEC and (**b**) OEC. In the TEC in which both sides of the core are connected to the two heat baths, we have less temperature difference, between the core and outer CNTs, rather than the OEC case.

Temperature profiles of CNTs in a given structure provide insightful data to understand thermal transport processes in vdW heterostructures (see Fig. 4). Due to the distinct difference between the interaction of two carbon atoms, (i) inside CNT with the strong covalent band, and (ii) the weak Lennard Jones interaction, we may have adjacent atoms in two different CNTs, having different temperatures. Figure 4 shows how the temperature profiles of outer CNTs and the core are different. Specifically for the case of OEC (Fig. 4), in which the left side of the core is disconnected from the hot bath, the temperature of the core can not reach the temperature of the bath, from the leads to a lower thermal conductance for OEC structures, as compared to the corresponding TEC cases.

**Figure 5:**
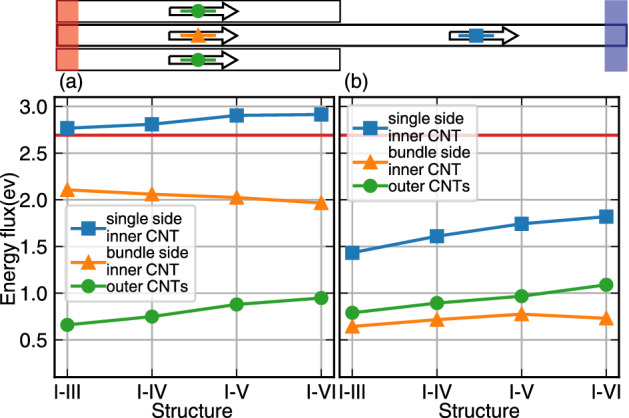
Heat current in the middle of single side (overall heat flux) and bundle side for the homogeneous bundles. (**a**) Represent the heat current for the TEC case, and (**b**) represent the heat current for the OEC case. The red line shows the value of heat flux in the (10,10) single CNT with the same simulation method with no outer CNTs. The horizontal axis shows the number of outer CNTs for example I–III represent three outer CNTs surrounding the inner CNT.

As shown in Fig. 5, there are different possible trajectories for heat to flow from the hot bath (left) to the cold one (right). If the core is mutually connected to both hot and cold heat baths (TEC), one major portion can flow directly from the core (orange triangle and blue rectangle in top Fig. 5), due to the high thermal conductance of the core. Simultaneously, the remaining portion transfer from outer CNTs (green circles) to the core as well. But, when just one side of the core is directly connected to the cold bath (OEC), all thermal phonons must transfer from outer CNTs to the core, where the accumulated total heat is received by the cold bath. Due to the low thermal conductance of vdW interaction (normal z-axes, radial direction between two CNTs) as compared to the high thermal conductance of covalent bonds (in the z-direction, alongside CNTs), the total conductance in the TEC system is higher than the corresponding OEC version. Mere interaction with outer CNTs in all cases can result in more phonon scattering in the core, which decreases the thermal conductance of the core. Nonetheless, in all cases, the entire heat must be transferred from left to right, and the flux magnitude in the blue rectangle zone (Fig. 5) will be the total steady flux for each case.

As precisely described by Rajabpour et al.^33^, the transport trajectory of heat is somehow complicated. Also in our studied cases, if we consider flow in the z direction, a part of flow will be transmitted through outer CNTs to the core and the rest inside the core. It means that for the left side of the system in Fig. 2a, at a given point in the z direction, there will be no thermal equilibrium, since the temperature in outer CNTs and the core are not the same (Fig. 4). But if we make an average over their temperature, a Kapitza like feature will be obtained. This discontinuity mimics the effect known as the Kapitza resistance at the interface which for both TEC and OEC can be calculated^34^ (next sections).

### The effect of shared vdW surface

To understand the contribution of outer CNTs to homogeneous cases (same diameter of CNTs), heat transport in four different structures have been simulated (Fig. 2a). In these cases, as described (see above), because the distance between each of the two adjacent outer CNTs is larger than the vdW equilibrium distance, each outer CNT may rotate around and/or slip over the core, which could lead to possible asymmetric structures (see Fig. 3). Thus a few atoms have been frozen to avoid all mechanical instabilities.

The heat flux for two sides of the systems (the middle of each side), for single side, or bundle side is shown in the Fig. 5. As it is seen, by increasing the number of outer CNTs, the total heat flux will also increase (blue line in Fig. 5), and for the case, I–VI largest heat flux magnitude is obtained. The variation of heat flux of the core in the bundle side (orange triangle) is not considered for different structures. Higher contribution of energy heat flux belongs to the outer CNTs increases *almost linearly* with the number of constructors. By comparing the right and left figures (Fig. 5), the major contribution of the core in the TEC case becomes more clear. Since the thermal conductance in a single CNT is too high, the amount of energy flux transferred from outer CNTs to the core is considerably lower than the energy flux transferred directly by the core, in the TEC case. But since the heat has no direct path to transfer from hot to cold bath in OEC structures, the energy flux of outer CNTs (green circles) and the core (orange triangle) in different cases are not much different from each other.

To make a conclusion on energy fluxes in these two cases, Fig. 5 gives an insightful understanding of the importance of covalent bonds for the whole process. We have a path constructed by covalent bands, through which the major amount of heat is transferred. But in the other case in which heat needs to pass a vdW junction, the contribution of the vdW shared surface is the leading parameter.

**Figure 6:**
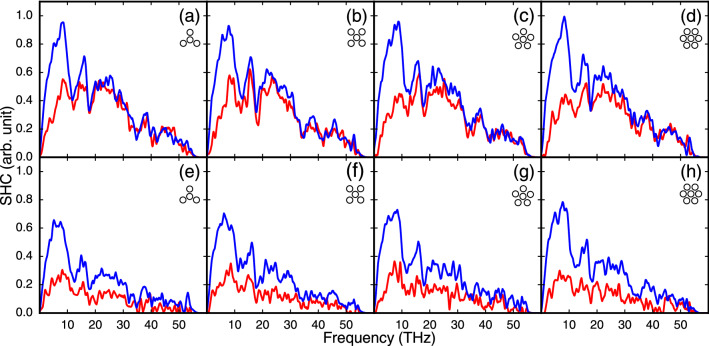
The SHC of the core in single side (red lines) and bundle side (blue lines). (**a–d**) Calculated for the TEC case, and (**e–h**) are the same for OEC ones. The small cartoon at the top right inside each figure depicts the structure. The major difference between the top and the bottom row is in the top row, the most significant difference between blue and red curves occurs at approximately 0–10 THz, but for the bottom row, in which just one end of the core is connected to the cold bath, there is a larger difference between red and blue curves for a wider range of frequencies.

To have more insightful data about the contribution of different phonons in heat transport, the SHC of a portion of atoms in the middle of right and left half of the core (orange triangle and blue square in Fig.  5 (top)) are calculated (is shown in FIG. 6). In FIG. 6, sub-figure a–d are the SHC of TEC case. As discussed above, the major part of the heat is directly transported through the core, and the effect of outer CNTs is most apparent in low frequencies (longer wavelengths). The red and blue curves in 0-10 THz interval, have meaningful differences as compared to the rest of the frequency domain, which means these modes transfer or are affected by corresponding modes in outer CNTs. But the SHC feature for the bottom row (e,f) is completely different. The difference between red and blue curves in OEC cases are not limited to a specific frequency interval and the red curves appear to the multiplied to a value less than one. The intensity of all modes is almost lower for red cases in the bottom row, indicating that there is no crucial difference between outer CNTs and the core. Since the heat has to pass through the core and outer CNTs Van der Waals interface, it seems all things are almost the same in this respect.

As can be seen in Fig. 6, the main trend of SHC does not vary by decreasing the number of outer CNTs, which is because there is no major difference in heat fluxes. The fact that the diameter of the core and outer CNTs are the same plays an important role in heat fluxes being equal, which brings about the effect of core curvature as discussed in the next section can play a role from this point of view, that we address this issue more precisely, somehow related to the curvature of core and outer CNTs (see below).

### The curvature effect

The focus of this section is devoted to the effect of the difference between the radius of the core and outer CNTs. To choose a unique or similar structure for all configurations, the most symmetric configurations have been considered. It means similar to the I–VI case in the previous part, that the distance between every two neighboring CNTs was the same and the distance between the core and each of them was also the same, the constructed structures of this section, have been set that way. That the distance between every two neighboring outer CNTs becomes similar to the I–VI ones, i.e., all distances between the core and outer CNTs and even neighboring outer CNTs are equal and called *vdW equilibrium distance* (Fig. 1).

**Figure 7:**
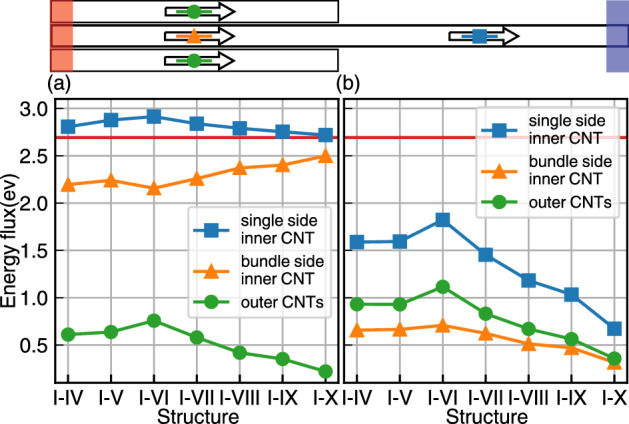
The heat flux for (**a**) TEC and (**b**) OEC heat fluxes for the OEC case. The blue square, the orange triangle, and the green circle correspond to the values for the middle of the core right side, the middle of the core on the bundle side, and the middle of outer CNTs as demonstrated in the top figure.

Using NEMD simulation heat transport, the steady thermal current for all cases is obtained (Fig. 7). Similar to the previous section, the flux is calculated at the middle of both half right and left sides, in the core (orange triangle and blue square) and outer CNTs (green circles).

At first, a comparison between TEC and OEC cases should be addressed, but it is important to emphasize that the energy flux of the I–VI structure is the maximum value in both cases [(a) and (b) in Fig. 7]. This result can so be called the first evidence of the importance of the homogeneity (same radius) or the effect of curvature difference in heat transport of vdW heterostructures.

To have a more insightful understanding of the whole process, similar to vdW shared surface cases, the heat current in two portions of atoms located in the middle of right (blue square) and left (orange triangle) half of the core, have been calculated (Fig. 7). As shown in this figure, the magnitude of the blue squares (the steady heat fluxes), for the TEC case, is at least 1 eV larger than corresponding cases in OEC. This indicates that the high thermal conductivity in the core can lead heat transport process, especially when its two ends are connected to hot and cold heat baths.

In contrast to the previous section in which by increasing the number of outer CNTs, the energy flux of outer CNTs increased, here, by increasing the number of outer CNTs, the energy flux has a decreasing feature, for systems that the number of outer CNTs is more than VI. Although these decrements vs increasing the number of outer CNTs can relate to the smaller diameter of outer CNTs, the increment in the number of tubes can be overwhelmed by the decrements in the vdW shared surface. In both *TEC* and *OEC* cases, the total heat flux (blue square), as well as the heat, passes from the outer CNTs to the core (green circles) have a maximum at the I–VI case.

It is needed to emphasize the main differences between the heat current for the core in all cases (orange triangle). For the TEC case, from I–IV to the I–VI structure, there is no considerable difference, but for from VII to the I–X case, the TEC structures have increasing feature, but the OEC ones have decreasing feature. Besides the fact that CNT with smaller diameters has lower thermal conductivity, due to the shorter phonon mean free path, thus this can suppress heat transport in outer CNTs, rather than corresponding cases with larger diameters. Thus, in TEC cases, the core plays the leading role in heat transport, and by decreasing the thermal conductivity of outer CNTs, the core contributes more significantly. But in OEC systems in which whole thermal energy has to be transferred through outer CNTs, they exhibit monotonic decrements from I–VI to I–X cases.

One other reason behind obtaining a maximum energy flux in the I–VI structure is related to the similar local curvature of two CNTs at their shared interface, that in which the phonons can be more easily radially transferred from one to the other. To have a more quantitative understanding of the claim, it is needed to investigate spectral density function adjacent CNTs as well as SHC.

**Figure 8:**
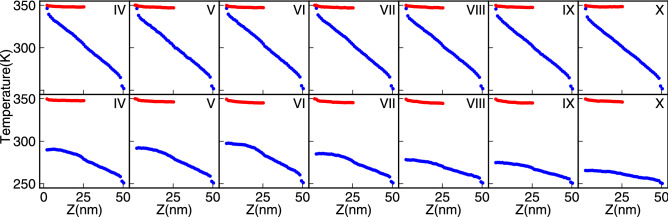
The temperature profiles of the core (blue) and outer CNTs (red) for the TEC (top row) and OEC (bottom row) structures. From the first (I–IV) to the last ( I–X) column, the curvature of outer CNTs becomes smaller.

Before dealing with spectral properties, it is important to have a precise description of the temperature profile of CNTs in each case. Similar to the previous section, in Fig. 8, the temperature profile of the core and outer CNTs are presented for both TEC (left) and OEC (right) cases. In bottom row cases that the hot bath is not directly connected to the cold one by covalent bonds, the buttle neck in phonon carrier is the svW shared surface between the core and outer CNTs. The lowest temperature difference between red and blue curves in OEC structures belongs to the I–VI case in which the nanotube curvatures are the same. In TEC cases, this feature is not clearly seen, because of the leading role of the core by which most of the heat transferred to the middle of the system. As it is seen, the temperature difference between the core and outer CNTs has a minimum value for the I–VI case. The same story about the leading role of the core in the TEC case is also valid.

**Figure 9:**
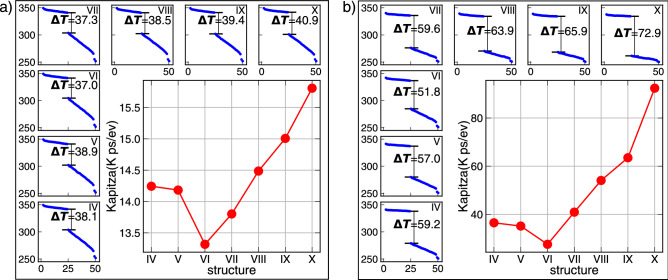
The average of the temperature of the core and outer CNTs along z direction (blue curves) for (**a**) TEC, and (**b**) OEC structures. By definition of the Kapitza like resistance (red curves), for both TEC (left) and OEC (right), the higher thermal conductance if I-VI structure can be concluded.

To characterize the difference between the temperature profile of the core and outer CNTs, it is possible to calculate a parameter similar to Kapitza resistance at the middle of both TEC and OEC structures. As previously discussed^33,34^, since there is no equilibration between the core and outer CNTs, it is not possible to assign a temperature to the system along z direction, but by averaging over the temperature of the core and outer CNTs, the same feature appears. In Fig. 9, the averaged temperature alongside z direction of TEC (left) and OEC (right) for all seven structures are presented. These Kapitza like parameters, which can be defined similarly to the Kapitza Resistance (${({\rm R_K}=\Delta {\rm T/J})}$) is also shown in the same figure. Very interestingly, this kapitza resistance can exhibit the lower resistance of the I–VI structure, corresponding to a higher thermal conductance rather than the other cases. As support on results presented in Fig. 7, the minimum Kapitza resistance corresponds to the maximum thermal conductance of the I–VI structure. As can be seen, by increasing the curvature difference between vary the higher Kapitza resistance is achieved.

**Figure 10:**
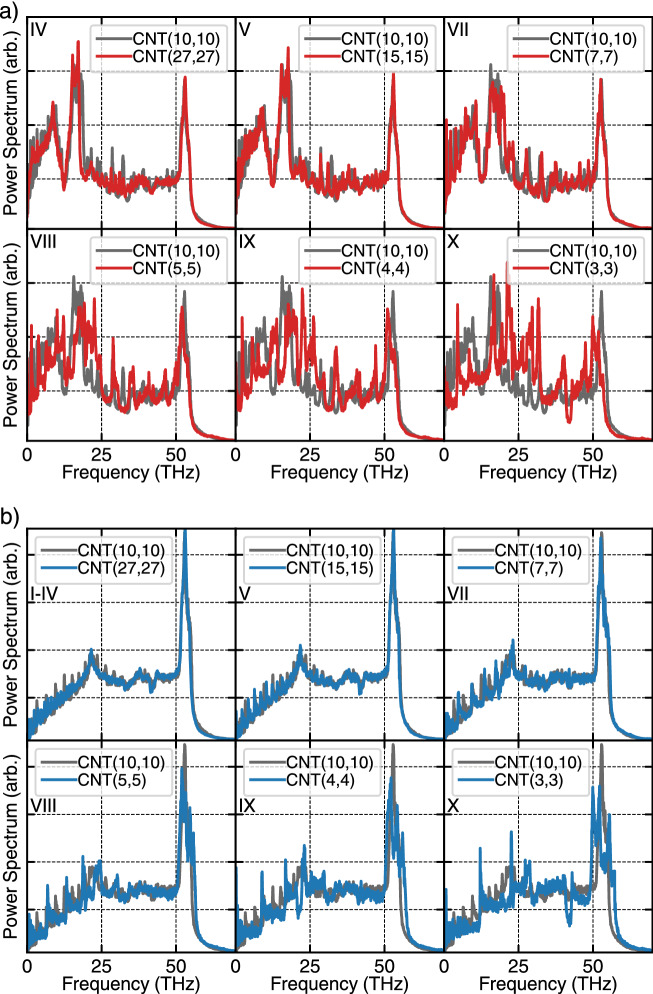
(**a**) Circumferential (in the off-axis direction of the CNT) power spectrum of outer CNTs (red lines) in comparison to core CNT(10,10) (gray lines) and (**b**) z-direction (in the axis direction of the CNT as shown in Fig. 1) power spectrum of Outer CNTs (blue lines) in comparison to the core (gray lines).

**Figure 11:**
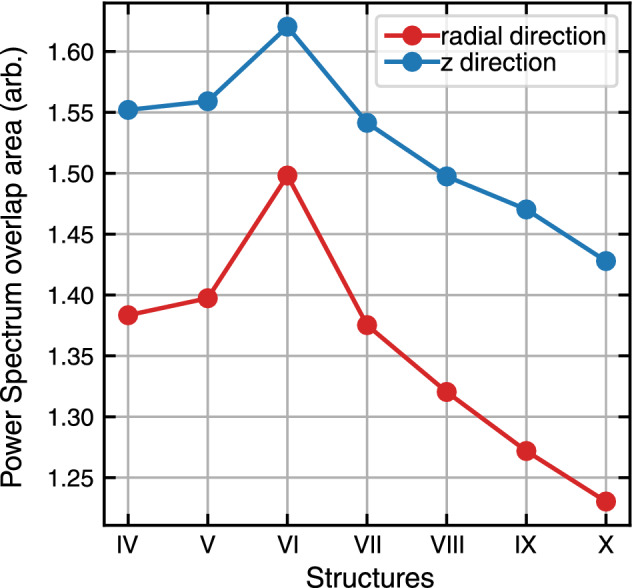
The overlap area of core CNT and outer CNTs power spectrum in each structure.

To have a better vision of phonon transfer, vibrational density of states or velocity power spectra of a portion of atoms in each CNT has been calculated (Fig. 10). In this figure, the gray lines are the radial velocity power spectrum of the (10,10) case, and the red lines are the same for outer CNTs. Depending on the radius of outer CNTs, as much as they are similar to the core, the velocity power spectra have less difference from the core ones (10,10). This is evident for less contribution of low frequency (acoustic) phonons, which means the radial (out of plane) modes transmit more difficult between core and outer CNTs. To have more insightful pictures from velocity power spectra, in Fig. 11, the overlap between every two curves of Fig. 10 calculated. The overlap magnitude has a maximum for the I–VI case and its trend is similar to the heat flux as shown in Fig. 7.

**Figure 12:**
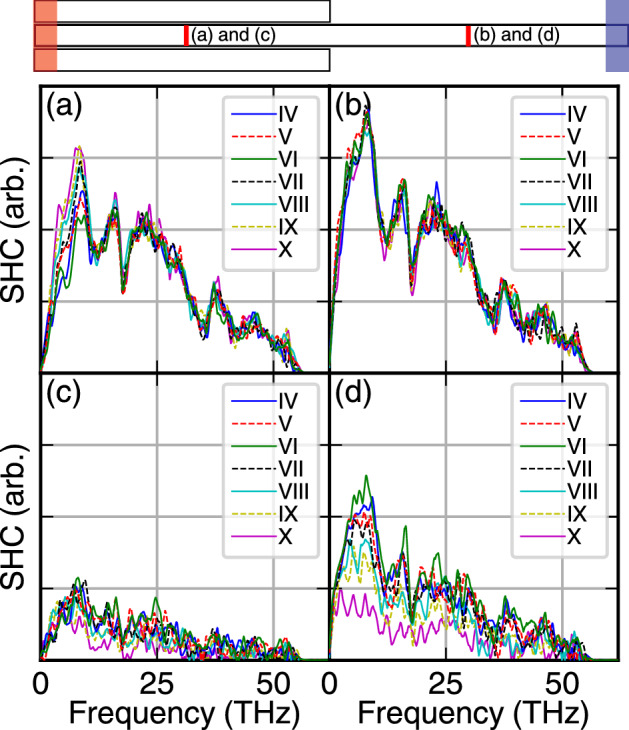
In the top figure, the two tiny red regions schematically show which portion of the core atoms has been considered for calculating Spectral Heat Conductance (SHC). In the top row (the TEC cases), the SHC of the middle portion of the core on the bundle side (**a**) and the single side (**b**) are presented. For OEC structures (bottom row), (**c,d**) exhibit the same calculated values for corresponding cases.

Similar to the previous case, SHC for these structures also provides nontrivial and insightful pictures. In Fig. 12a,b are the SHC for the single and bundle side of the core, for the TEC structure, respectively. Since the core is connected to both heat baths, we do not expect a serious deviation between the left and right sides of each case. Just small deviation in low frequencies (long wavelengths) corresponds to higher interaction of out of plain phonons. This difference is mostly located in 0–10 THz frequencies. The collapse of all curves over almost one curve [side SHC (Fig. 12a)], is another insight into the fact that in all structures the major part of the heat is not influenced by outer CNTs, and energy flux passes through the core. For Fig. 12c,d cases, the story is completely different. The SHC of the single side is almost present an accumulation of phonon carriers pass through shared vdW surfaces. Since the core CNT is not connected to the hot heat bath, the power spectral density has been considerably suppressed. Similar to previous pictures, the highest magnitude of spectra belongs to I–VI, that vibrational modes are the same between the core and outer CNTs. It is worth to mention that the lowest SHC belongs to a higher difference between the curvature of the core and outer CNTs, corresponding to the lowest overlap magnitudes in the studied cases (Fig. 11).

## Conclusions

In this paper, thermal transport in a CNT bundle, with different varieties of consisting elements and possible structures, such as the number of outer CNTs in the bundle, and the difference between the radius of outer CNTs and the core have been investigated. These structures cover all common local structures in possible given vdW heterostructures, such as a nanotube forest and/or an assembled nanotube structure. To cover all kinds of possible transport mechanisms, the OEC and TEC cases have been included in simulations, to show when there is no completely covalent connected path between the hot and cold sides of the system.

To emphasize how the vdW contact area or vdW shared surface affects on heat transport, four different structures of homogeneous CNT bundles (bundles with the same CNTs) have been studied (Fig. 2a). In OEC cases in which any amount of transferred heat from hot (left) to cold (right) bath has to pass through the vdW interface, i.e. the area that outer CNTs match to the core, by increasing the vdW interface (from I–III to I–VI), the increment of thermal conductance is clearly seen (Fig. 5). In these structures the radius of the outer and the core are the same, there is no curvature effect, thus just increasing the interface area that controls total heat flux (flux of the core at the single side). But, more interestingly, in the TEC homogeneous system, in which there are two different paths for heat to transport from the left to right sides of the system, the core that consisted of carbon-carbon covalent bonds, takes care of a considerable amount of thermal conductance. But for the remaining amount of the transfers that pass from outer CNTs to the core, we have a complicated feature. These vdW interactions between outer CNTs and the core lead to a negative effect on the individual thermal conductance of the core (triangles in Fig. 5a), that it can be considered as the result of inter-layer vdW interactions which lead more phonon scattering; this can be considered as the source of small decrement. But, the total conductance, the combination of outer CNTs to the core plus intrinsic transport by the core, shows increasing thermal conductance from I–III to I–VI case.

One major result of the paper was to clarify how a covalent direct path between hot and cold heat baths, affects the feature of heat transport. This understanding came from the comparison of the TEC and OEC structures. But the other key parameter in heat transport is the curvature difference between outer CNTs and the core. Due to the mismatch between the vibrational density of states of CNTs with different radii (curvatures), this also needs to be considered for understanding the transport mechanism in these heterostructures. If the radius of outer CNTs are larger, or smaller than the core, the maximum number of outer CNTs changes. Thus, to make a clear overview of the whole process, all cases have been studied. For a larger outer CNT radius, the number of outer CNTs is smaller, but the interface is larger. But, this difference leads to smaller thermal conductance in the system. For CNTs with smaller radii, the outer CNTs are more than homogeneous case, but the vibrational difference leads to more thermal resistance in the system. This lead to a clear conclusion: if we consider the system, similar to two side system with an artificial kapitza resistance between the left and right side, the lower resistance belongs to the homogeneous case, I–VI, and the rest of the case, with larger CNT radii (IV and V) and even smaller ones (VII, VIII, IX, and X), thermal conductance of the system is smaller.

The heat flux in the bundled CNTs is maximum, by increasing the number of outer CNTs to 6 and decreasing their curvature or radius difference. By simulating different systems with a different number of outer CNTs with a variety of radius, this result has been concluded. Frequency-dependent analysis, including velocity power spectra as well as SHC, supported this conclusion.

These results provide an insightful understanding of heat transport in vdW nanostructures, more specifically important in nanoelectronic devices as well as systems in which the asymmetry of transport has crucial importance.

## Supplementary Information

Supplementary Information.

## Data Availability

The data sets used and/or analysed during the current study available from the corresponding author on reasonable request.
