# Supplementary material for: Curvature and van der Waals interface effects on thermal transport in carbon nanotube bundles
Source: Sci Rep. 2022 Nov 14;12:19531. doi: 10.1038/s41598-022-22641-y (PMC9663422; doi:10.1038/s41598-022-22641-y)
Supplement: Supplementary file 1 — Supplementary Information. [file 41598_2022_22641_MOESM1_ESM.docx]

# Supplemental Materials

### Curvature and Van der Waals interface effects on thermal transport in Carbon Nanotube Bundles

Mostafa Valadkhani,^1^ Shunda Chen,^2^ Farshad Kowsary,^1^ GiulianoBenenti,^3,4,5^ Giulio Casati,^3,6^ and S. Mehdi Vaez Allaei^7, 8, ∗^

1 School of Mechanical Engineering, College of Engineering, University of Tehran, Tehran, Iran

2 Department of Civil and Environmental Engineering, George Washington University, Washington, DC 20052

3 Center for Nonlinear and Complex Systems, Dipartimento di Scienza e Alta Tecnologia, Universit`a degli Studi dell’Insubria, via Valleggio 11, 22100 Como, Italy

4 Istituto Nazionale di Fisica Nucleare, Sezione di Milano, via Celoria 16, 20133 Milano, Italy

5 NEST, Istituto Nanoscienze-CNR, Piazza S. Silvestro 12, 56127 Pisa, Italy

6 International Institute of Physics, Federal University of Rio Grande do Norte, Campus Universit ́ario - Lagoa Nova, CP. 1613, Natal, Rio Grande Do Norte 59078-970, Brazil

7 Department of Physics, University of Tehran, Tehran 14395-547, Iran

8 School of Nano Science, Institute for Research in Fundamental Sciences (IPM), Tehran 19395-5531, Iran

*corresponding author, email: smvaez@ut.ac.ir

To support the independence of our main findings from the covalent C-C potential, we performed additional simulations with the reoptimized Tersoff potential (PRB 81, 205441 (2010)) that was also studied in Phys. Rev. Materials 1, 056001 (2017). Specifically, we performed additional simulations of the I-V, I-VI, I-VII, and I-VIII structures for the TEC case and additional simulations of the I-V, I-VI, I-VII, and I-VIII structures for the OEC case, using the reoptimized Tersoff potential (PRB 81, 205441 (2010)) (with the same van der waals (LJ) parameters used in the AIREBO potential).

The data of new simulations are shown in the following figures. By comparing the results obtained by the reoptimized Tersoff potential to the results obtained by AIREBO potential, one can see that indeed the reoptimized Tersoff potential does give the same trends as the AIREBO potential for both TEC and OEC cases, that I-VI structure has the lowest Kapitza resistance and highest thermal conductance. Our additional simulations strongly confirm that the main findings (trends) reported in our manuscript are robust and do not depend on the choice of the covalent C-C potentials.


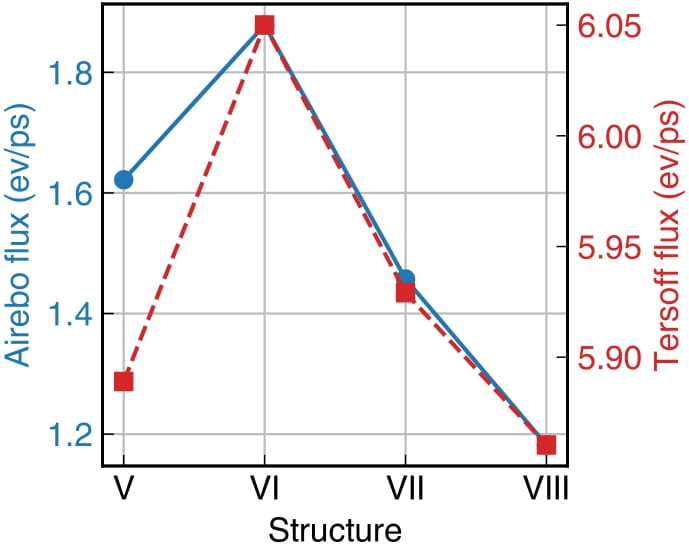


FIG. S1. Overall heat flux of I-V, I-VI, I-VII, and I-VIII structures for the TEC case.


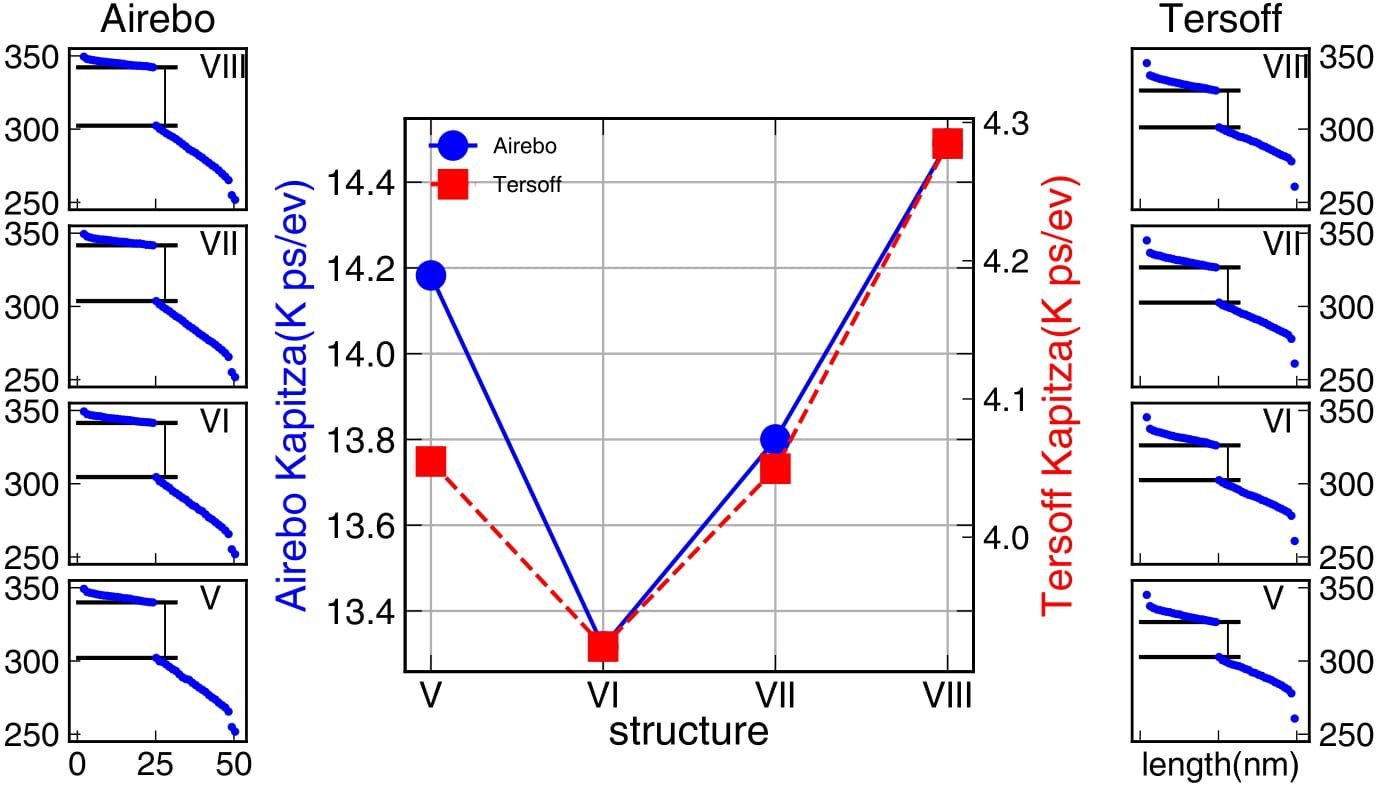


FIG. S2. Temperature profiles and Kapitza resistance of I-V, I-VI, I-VII, and I-VIII structures for the TEC case.


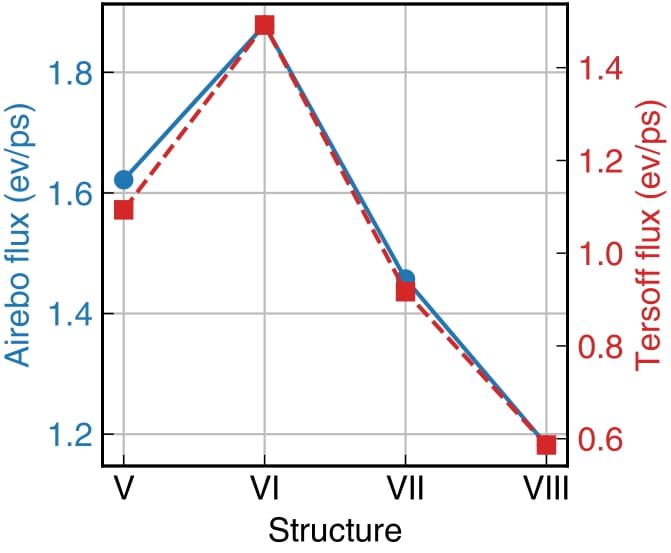


FIG. S3. Overall heat flux of the I-V, I-VI, I-VII, and I-VIII structures for the OEC case.


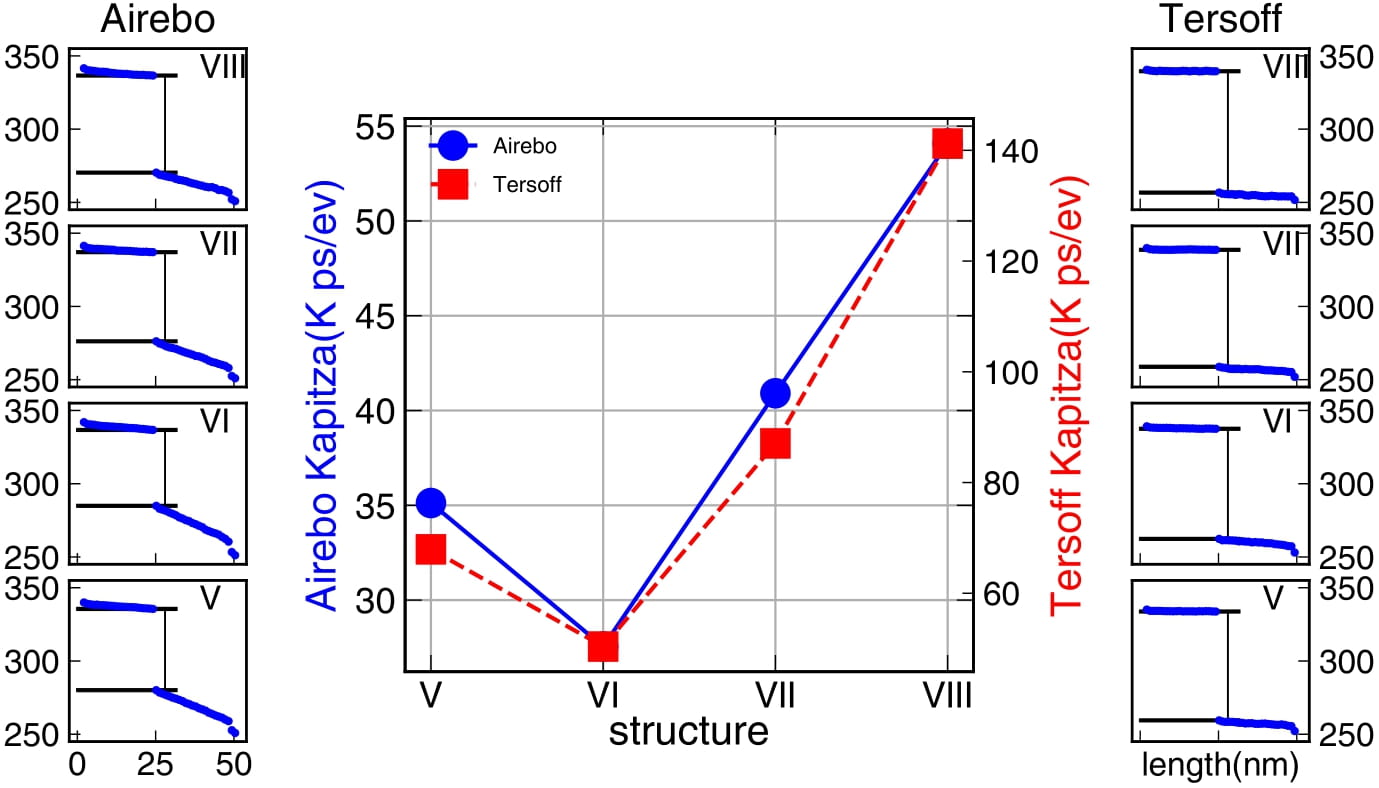


FIG. S4. Temperature profiles and Kapitza resistance of the I-V, I-VI, I-VII, and I-VIII structures for the OEC case .

As expected, the values of the heat flux and Kapitza resistance obtained by the reoptimized Tersoff potential are not exactly the same as those obtained by AIREBO potential, but the trends are in very good agreement (see Figs. S1-S4). It is interesting that for the TEC case the values of heat flux obtained by Tersoff potential are about three times larger than those obtained by using Airebo potential, while for the OEC case the values of heat flux obtained by Tersoff potential are very close to those obtained by using Airebo potential. This is because for the OEC case the total heat flux has to pass through the van der waals interaction between core and outer CNTs, and the simulations with Tersoff potential and AIREBO potential have the same van der waals parameters. In FIG. 4, the higher temperature gaps in the simulations with Tersoff potential are due to the higher thermal conductivity of CNTs with Tersoff potentials. The same trends reproduced by using the reoptimized Tersoff potential strongly suggest that the main findings (trends) reported in our manuscript are robust and independent on the choice of the covalent C-C potentials.
